# Editorial for the Special Issue: Thermophiles and Thermozymes

**DOI:** 10.3390/microorganisms7030062

**Published:** 2019-02-27

**Authors:** María-Isabel González-Siso

**Affiliations:** Grupo EXPRELA, Centro de Investigacións Científicas Avanzadas (CICA), Facultade de Ciencias, Universidade da Coruña, 15071 A Coruña, Spain; isabel.gsiso@udc.es

Heat-loving microorganisms or thermophiles arouse noticeable scientific interest nowadays, not only with the aim to elucidate the mystery of life at high temperatures, but also due to the huge field of biotechnological applications of the enzymes they produce or thermozymes, able to function under industrial harsh conditions. 

This Special Issue contains nine papers that study diverse aspects of thermophiles biology and their enzymes.

Two research articles deal with the genomics of thermophilic microorganisms. Blesa et al. [1] describe the characterization of active and inactive insertion sequences spanning the genus *Thermus*. This work represents an interesting contribution both to the construction of new genetic engineering tools and to the knowledge of the genomic plasticity and capacity of adaptation of thermophilic microorganisms. Schouw et al. [2] analyze the genome of a fermentative new strain isolated from a deep-sea hydrothermal vent, which was reassigned to the genus *Vallitalea* and designated *V. guaymasensis* strain L81, showing interesting features for industrial application. Furthermore, the potential of these marine ecosystems for the bioprospection of new enzymes and antimicrobials is revealed in this work.

Four other research articles deal with different aspects of thermozymes. J.M. González [3] presents a structural analysis of substrate tunnels in two types of enzymes (with low and high tunneling) from microorganisms living optimally at 15 °C to 100 °C. Molecular tunnel dimensions are reduced with increasing optimum growth temperatures, minimizing unnecessary spaces within the molecule. From this work, molecular channeling appears as a mechanism that helps to understand how thermophiles are adapted to live under high temperatures. Álvarez-Cao et al. [4], using the lipase LipE from *Candida rugosa*, show that oligomerization-dimerization is another structural feature that causes protein stabilization against temperature and pH, also expanding substrate specificity on soluble substrates. Domain swapping is the mechanism proposed to explain LipE homodimerization. Bibra et al. [5] report the statistical optimization of the production of a thermostable xylanase from a *Geobacillus* sp. strain isolated from a gold mine, its comparison (favorable) with commercial counterparts for lignocellulosic biomass hydrolysis, and the use of the strain in co-culture for ethanol fermentation of the biomass. Gomri et al. [6] depict the characterization of a new acid protease produced extracellularly by a thermophilic bacterium that was isolated from an Algerian hot spring and affiliated with *Brevibacillus thermoruber* species. The purified 32-F38 protease resulted to be thermostable and highly stable in the presence of different detergents and solvents, suggesting potential biotechnological applications.

Three reviews complete this monograph. The one by Hori et al. [7] is a comprehensive study about modified nucleosides in tRNA and tRNA modification enzymes from thermophiles, in view of strategies to stabilize tRNA structures including RNA-binding proteins and polyamines. The one by Finch and Kim [8] focuses on the application of thermophilic proteins as scaffolds for protein engineering, proposed due to their robustness and evolvability, without forgetting the trade-off between protein activity and stability. Finally, the one by Escuder-Rodríguez et al. [9] introduces the metagenomics approach, in this case for the search of thermostable cellulases, a complex group of enzymes which is here unraveled.

Altogether these papers allow us to go one step forward to explain how the thermophilic microorganisms and their enzymes are stable and functional at high temperatures, and to envisage new biotechnological applications and fields for bioprospection. 
